# Optimisation of Through-Thickness Embedding Location of Fibre Bragg Grating Sensor in CFRP for Impact Damage Detection

**DOI:** 10.3390/polym13183078

**Published:** 2021-09-12

**Authors:** Helena Rocha, Ugo Lafont, João P. Nunes

**Affiliations:** 1 Institute for Polymers and Composites, University of Minho, 4804-533 Guimarães, Portugal; jpn@dep.uminho.pt; 2 PIEP–Innovation in Polymer Engineering, University of Minho, 4800-058 Guimarães, Portugal; 3 European Space Research and Technology Centre, European Space Agency, 2201 AZ Noordwjik, The Netherlands; Ugo.Lafont@esa.int

**Keywords:** carbon fibre reinforced polymer, composite materials, barely visible impact damage, fibre Bragg grating sensor, low-velocity impact, impact resistance, impact response, structural health monitoring

## Abstract

Aerospace composites are susceptible to barely visible impact damage (BVID) produced by low-velocity-impact (LVI) events. Fibre Bragg grating (FBG) sensors can detect BVID, but often FBG sensors are embedded in the mid-plan, where residual strains produced by impact damage are lower, leading to an undervaluation of the damage severity. This study compares the residual strains produced by LVI events measured by FBG embedded at the mid-plan and other through-thickness locations of carbon fibre reinforced polymer (CFRP) composites. The instrumented laminates were subjected to multiple low-velocity impacts while the FBG signals were acquired. The FBG sensor measurements allowed not only for the residual strain after damage to be measured, but also for a strain peak at the time of impact to be detected, which is an important feature to identify the nature and presence of BVID in real-life applications. The results allowed an adequate optical fibre (OF) embedding location to be selected for BVID detection. The effect of small- and large-diameter OF on the impact resistance of the CFRP was compared.

## 1. Introduction

The aircraft and aerospace industries have shown an increased demand for fibre reinforced polymer (FRP) composites in the past few decades, aiming to replace metallic structures. FRP composites allow the mechanical performance to be increased and the weight ratios of structural parts to be decreased, which, consequently, allows the initial purpose of reducing fuel consumption, carbon dioxide emissions, and costs to be attained [1]. Although composite materials present great mechanical properties and lightweighting characteristics, their failure behaviour is as yet difficult to foresee, as a combination of defects, such as fibre breakage and/or fibre misalignment, matrix micro- or macro-cracking, stress and material discontinuities, matrix/fibre debonding, and delaminations, can be found simultaneously [2]. Aeronautic composites are prone to BVID, produced by LVI, which can produce front-face damage, but also internal transverse cracks, delaminations, and fibre breakage that can go undetected during inspection operations [3].

The adoption of structural health monitoring (SHM) systems for damage detection is crucial to guarantee that structures are intervened upon after damage, which may possibly contribute to an extended structure lifetime [4]. SHM systems resort to surface-mounted or embedded sensors to provide continuous data that will be decisive to performing inspection and repair operations focused on the damaged areas, preventing scheduled periodic inspections [1]. FBG sensors are OF-based sensors. FBG sensors are deemed a matured technology for SHM [5], able to monitor LVI damage both under static and dynamic conditions [6]. However, for a given impact energy, damage detectability depends on the distance between FBG and impact location, whereas damage extent is dependent on the material properties and structure of the composite.

An FBG sensor is a narrowband reflector consisting of a series of periodic gratings with a refractive index different than that of the OF core material, and working as a mirror of the Bragg central wavelength (*λ_B_*) [1,7]. *λ_B_* is dependent on the grating effective refractive index, *η_eff_*, and grating period, Λ, as stated in Equation (1). A local deformation will produce a change in the grating period, producing a variation in the reflected *λ_B_*, allowing the induced strain to be detected [4]. FBG sensors present the advantage of being intrinsic sensing elements, where the recorded signal is taken directly in wavelength, facilitating wavelength division multiplexing, wherein several sensors can be inscribed in the same OF [8].

(1) $$\lambda_{B}=2\eta_{eff}\Lambda$$

Temperature also affects *λ_B_*, requiring compensation for thermal strains if only mechanical strain is to be measured [9]. Thermal compensation can be accomplished by resorting to an additional strain-free FBG sensor, given that all FBG sensors are exposed to the same temperature, or by encapsulating an additional FBG sensor inside a capillary, where the *λ_B_* shift is solely affected by the temperature effect [4].

The embedding method, location, and placement of optical fibre-based sensors in polymer composite materials should be thoroughly considered to reduce the impairment of mechanical properties. Theoretically, the modulus and strength deterioration depend on the angle between the OF and adjacent reinforcing fibres, laminate thickness, and OF diameter and protective coating. The higher the angle between the OF and adjacent reinforcing fibres, the higher the degradation of the mechanical properties. Yet it will mostly only be significant for structures with a high density of OF. Placing the OF parallel to the reinforcement fibres will generally result in a more uniform consolidation around the OF, with minor defects and reduced weakening of the composite mechanical properties [7]. The different diameter of OF and carbon or glass fibre reinforcements, with the former being about 10–15 times greater than the latter, may create some discontinuities [3]. Small-diameter optical fibres (SDOF) are reported to be an applicable solution, whereas large-diameter optical fibres (LDOF) promote poor consolidation around the reinforcing carbon fibre (CF), producing matrix-rich areas [10,11].

Often OF-based sensors are embedded the in the composite mid-plan, which is usually not where damage is created, which may lead to undervalued damage extent. To the authors’ knowledge, there has not been any research done on the through-thickness embedding location of FBG sensors and its influence on damage-extent evaluation. This paper studies the residual strain measured by FBG sensors at different through-thickness locations of CFRP composites subjected to LVI tests. Laminates made of epoxy and unidirectional CF fabric were produced by a vacuum-assisted resin infusion (VARI) process. Because impact resistance is highly dependent on the materials and layup structure, initial drop-weight impact tests were performed on non-instrumented reference specimens to identify the range of impact energies that ensure BVID. The instrumented samples were then exposed to a number of low-velocity drop-weight impacts with the selected range of impact energies while FBG signals were acquired to evaluate residual strain dependency on the OF through-thickness location. The distinct composite impact response, when impacted with different impact energies, was revealed by the measured residual strain. Besides measuring the residual strain produced by the LVI events, the used setup enabled the detection of the LVI event itself. This is particularly important for the implementation of SHM systems on real CFRP structures for accurate damage-severity assessment. Non-destructive analyses were used to evaluate the generated damage and validate the suitability of the FBG location to detect damage. To mitigate the impairment of OF embedment on the mechanical properties, the OF was placed parallel to the direction of the adjacent reinforcing fibres. Moreover, this work compared the influence of regular OF and small-diameter OF.

## 2. Materials and Methods

### 2.1. Materials

The epoxy Biresin^®^ CR83 resin and CH83-6 hardener from Sika AG, Switzerland, and unidirectional CF fabric 350UT from Toray Industries, Inc., Japão, with an areal weight of 340 g/m^2^ and thickness of 0.67 ± 0.10 mm were used to produce laminates. Ten layers of symmetric CFRP were produced by VARI with layup sequence [0/0/45/90/45] s.

**Figure 1:**
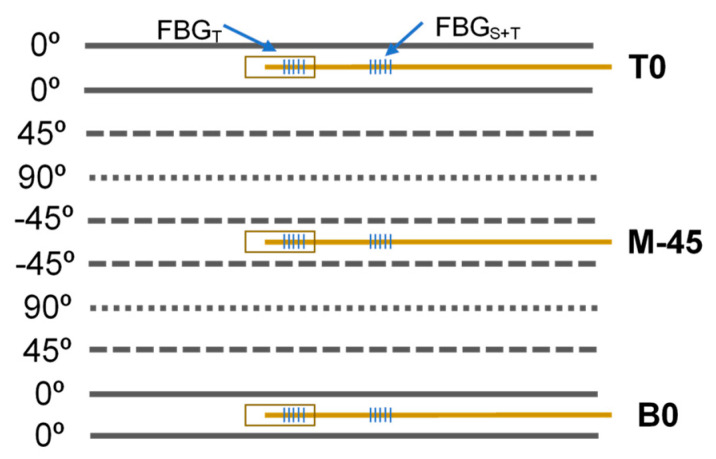
Schematic of optical fibre through−thickness embedding locations.

Optical fibres of different diameters were used: SDOF with an outer diameter of about 70 µm and LDOF with an outer diameter of about 150 µm. Each fibre had one strain and temperature-sensitive FBG sensor (FBG_S+T_) and one temperature-sensitive FBG sensor (FBG_T_). The LDOF, purchased from HBM FiberSensing S.A., Portugal, had *λ_B_* at 1540 and 1555 nm for the FBG_S+T_ and FBG_T_, respectively. The FBG_T_ was encapsulated into a 4 cm-long capillary with a 900 μm diameter and was 12.5 cm away from the FBG_S+T_. Additionally, LDOF with a single FBG_S+T_ with *λ_B_* at 1550 nm was used. The SDOF, purchased from Technica, USA, had a FBG_S+T_ with *λ_B_* at 1540 nm and FBG_T_ with *λ_B_* at 1550 nm, which were 2.5 cm apart from each other. The FBG_T_ was encapsulated inside a 4 cm-long fused silica capillary with an external diameter of about 363 μm. As the FBG_T_ was loose and could move freely inside the capillary, which was closed at both ends to prevent resin from going inside during the resin infusion, its *λ_B_* variation was only dependent on the effect of temperature on the coefficient of thermal expansion (CTE) and thermo-optic coefficient of the OF. On the other hand, FBG_S+T_ sensors were in direct contact with the laminate material, with the *λ_B_* variation being dependent on the CTE and thermo-optic coefficient of the OF itself and on the laminate mechanical strain. By subtracting the change in *λ_B_* measured on the temperature-sensitive FBG sensor, Δ*λ_T_*, the portion of *λ_B_* change due to the effect of mechanical strain alone on the FBG_S+T_, Δ*λ_S_*, could be calculated. Strain and temperature changes were calculated according to Equations (2) and (3).

(2) $$\Delta \varepsilon ={\Delta \lambda }_{S}\times S_{\varepsilon }$$

(3) $$\Delta T={\Delta \lambda }_{T}\times S_{T}$$

where *S_ε_* and *S_T_* are the strain and temperature sensitivities [12], at 1.2 pm/με and 10 pm/°C, respectively. The FBG_T_ sensors were used for cure monitoring studies, which are reported elsewhere [13]. Due to the different lengths of the SDOF and LDOF, laminate specimens were produced with different dimensions. The laminates with embedded SDOF presented dimensions of 150 × 100 × 3.3 mm, and the laminates with embedded LDOF presented dimensions of 190 × 100 × 3.3 mm. The locations of the OF were chosen so that they were embedded in between two layers of reinforcing fabric with the same direction, resulting in minimal defects and least impairment of the mechanical properties of the composite. Each specimen had only one OF in one of the positions shown in Figure 1. Location M-45 was the mid-plan with the OF placed in between two CF layers oriented in the −45° direction. Location T0 was the closest to the top impact surface and location B0 was the furthest position from the impact surface, where frequent fibre breakage was observed for this thin laminate. In both locations, OF was placed in between two layers oriented at 0°.

### 2.2. CFRP Manufacturing

**Figure 2:**
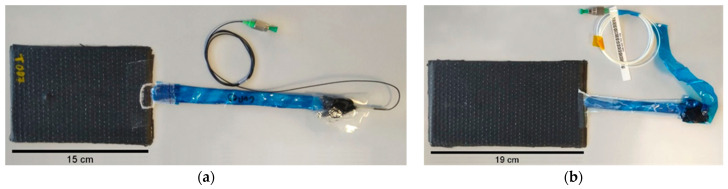
Composite specimens with embedded (**a**) SDOF and (**b**) LDOF for low-velocity drop-weight impact testing.

As the composite laminates were produced by the VARI process, the OF was first protected using flexible foam or thin film and adhesive tape. Peel ply layers were placed underneath and on top of the OF, wrapping to ease demoulding. A glass plate was used as the bottom mould where the release agent was applied. Layers of CF fabric and OF were stacked with the desired orientation. Peel ply and flow enhancement medium were placed on top of the fabrics and inlet and outlet resin flow lines were installed. The vacuum bag was sealed, and vacuum was applied. Resin and hardener were prepared according to the manufacturer instructions with a weight ratio of 100%/30%, respectively. The laminate was initially left to cure at room temperature under vacuum for at least 40 h and was later demoulded and post cured at 70 °C for 8 h, as indicated by the manufacturer. Figure 2 shows pictures of produced samples with embedded SDOF (a) and LDOF (b), where it is possible to see the OF protecting materials used inside the vacuum bag.

### 2.3. Impact Tests

**Figure 3:**
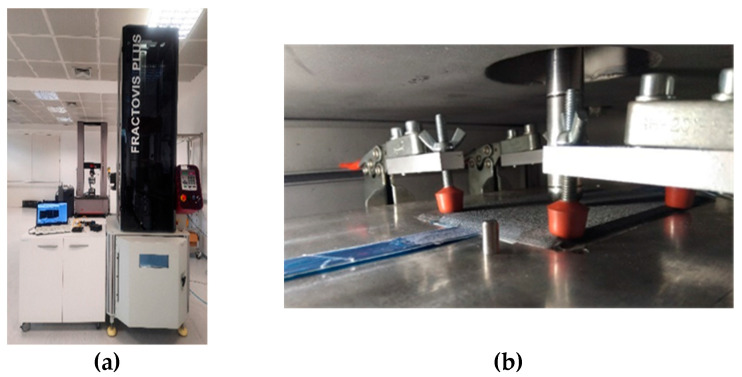
Drop-weight impact test setup: (**a**) OF interrogation equipment and impact testing machine; (**b**) impact support fixture with toggle clamps and impactor.

Drop-weight impact tests were performed according to the ASTM D7136 standard [14] using Fractovis Plus impact testing equipment from CEAST, Italy, (Figure 3). A reference laminate without sensors was produced and cut into samples of 150 × 100 mm. Three specimens were exposed to different impact energies of 13.1, 15.1, 17.5, 20.0, 25.0, 30.0 and 40.1 J. The different impact energies were achieved by varying the vertical position of the impactor head between 264 and 810 mm. The impactor had a hemispheric shape with a 20 mm diameter and a mass of 5.045 Kg. The impactor contact force on the specimen surface was recorded against time for each impact. From there, the impactor velocity *v*(*t*), displacement *δ*(*t*), and absorbed impact energy *E_a_*(*t*), as a function of time, were calculated following Equations (4), (5), and (6), respectively [14].

(4) $$v\left(t\right)=v_{i}+gt-\int_{0}^{t}\frac{F\left(t\right)}{m}dt$$

where *v_i_* is the initial impactor velocity, *g* is the gravitational acceleration, *F*(*t*) is the measured load at time *t*, and *m* is the total drop mass.

(5) $$\delta \left(t\right)=\delta_{0}+v_{i}t+\frac{gt^{2}}{2}-\int_{0}^{t}\int_{0}^{t}\frac{F\left(t\right)}{m}dtdt$$

where *δ*_0_ is the impactor displacement from the reference location.

(6) $$E_{a}\left(t\right)=\frac{m\left(v_{i}^{2}-v{\left(t\right)}^{2}\right)}{2}+mg\delta \left(t\right)$$

**Figure 4:**
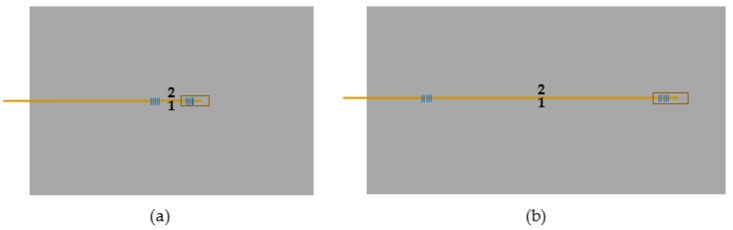
Schematic of approximate impact locations on specimens containing (**a**) SDOF and (**b**) LDOF. Numbers 1 and 2 represent the locations of the first and second impacts, respectively.

These initial impact results allowed the range of impact energy to be selected that undoubtedly and consistently produced BVID on the composite laminate in the form of subtle bumps on the back surface, but that could still go unnoticed in real-life applications where impact event existence and location are unknown, to be used in the forthcoming tests of instrumented composites. All the composite samples with embedded OF were subject to an initial impact with an energy of 30.0 J and a second one with an energy of 20.0 J. The first impact was about 1 cm away from each FBG sensor in the specimens with SDOF and about 6 cm away from each FBG sensor in the specimens with LDOF. The second impact location was about 1 cm away from first impact site. Approximate impact locations are schematically represented in Figure 4.

### 2.4. Non-Destructive Phased Array Ultrasonics

Non-destructive testing through phased array ultrasonics was conducted on the instrumented samples prior to and after impact testing to evaluate the induced damage and validate the OF measurements. The analyses used a Prisma ultrasonic flaw detector from Sonatest, United Kingdom, with a 5 MHz probe, 50 mm wide. The specimens were immersed in water and scans were performed at 100 mm/min.

### 2.5. Strain Monitoring

The DL-BP1 1501A super-luminescent LED source and I-MON 256 USB High Speed interrogation monitor with a 0.5 pm wavelength fit resolution from Ibsen Photonics, Denmark, were used to record the FBG data during impact testing. Signal acquisition was taken at the maximum measurement frequency of 6000 Hz for impact detection.

## 3. Results

### 3.1. Selection of Impact Energies for Production of Barely Visible Impact Damage

**Figure 5:**
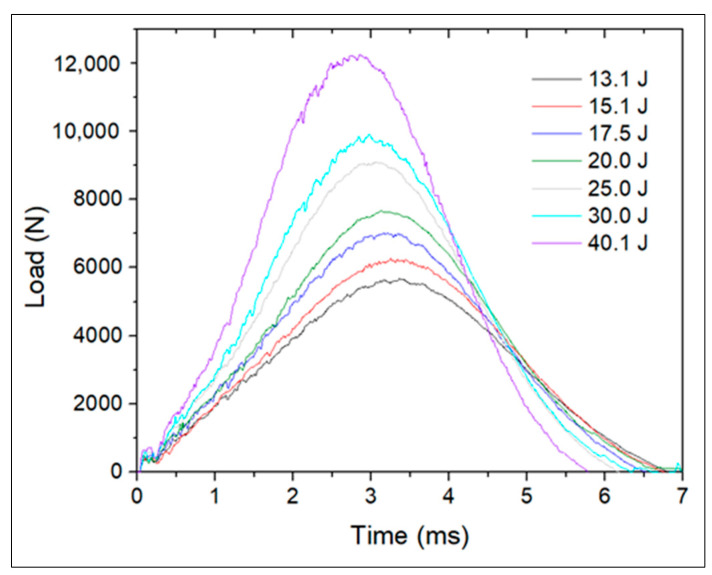
Impactor contact force for each impact energy tested on the reference CFRP.

The damage imposed on non-instrumented reference specimens during drop-weight impact testing was evaluated to select adequate impact energies that produce BVID. The recorded impact contact loads are plotted in Figure 5 and the averages of absorbed energy and maximum impactor contact force of three specimens for each level of energy are presented in Table 1.

The maximum impact force increased with impact energy. For impact energies between 13.1 and 30.0 J, the absorbed energy did not change significantly. Although there was an increase of absorbed energy of roughly 9% for impacts with 40.1 J, the load vs. time graph in Figure 5 does not reveal severe damage. Yet, the samples exposed to impact energies of 40.1 J showed lower contact time, revealing higher stiffness.

All the samples presented an indentation at the impact spot, some of them with small matrix cracks transverse to the CF in the top layer. Some samples tested with impact energies between 13.1 and 17.5 J showed a very smooth and hardly noticeable bump on the back surface, whereas samples exposed to impact energies between 20.0 and 30.0 J presented progressively more noticeable bumps on the back surface, but that could still go unnoticed in real-life applications where impact existence and location are unknown. The bump produced on samples exposed to 40.1 J would likely be detected in an attentive inspection. Hence, impact energies between 20.0 and 30.0 J were selected for tests on instrumented laminates. Each sample was first exposed to an impact event of 30.0 J to ensure that BVID was consistently imposed on the sample and that the FBG sensor could detect it, and to a second impact event of lower impact energy of 20.0 J, but that could still produce BVID.

### 3.2. Low-Velocity-Impact Monitoring in CFRP Laminates with Embedded FBG Sensors

**Figure 6:**
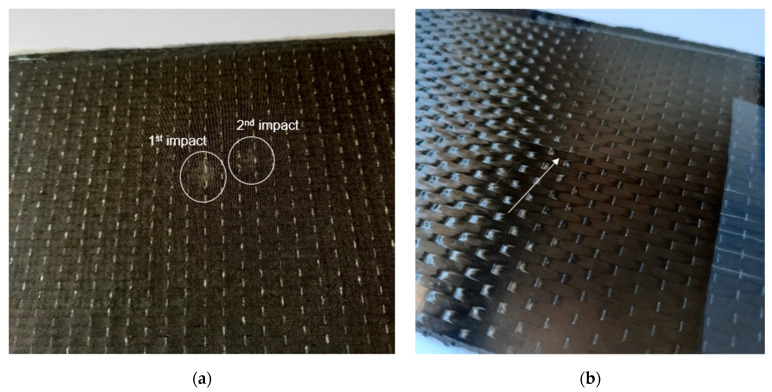
Example of (**a**) indentation marks on the top surface resulting from the first and second impact events, and of (**b**) small bump on the bottom surface.

Different FBG through-thickness locations were evaluated for BVID detection. BVID was imposed by drop-weight impact testing with the energy range selected in Section 3.1. Each sample was subjected to a first and second impact with energies of 30.0 and 20.0 J, respectively. The majority of damage was observed close to the bottom surface, where small bumps were visible (Figure 6b), as also observed in the reference laminates. This was expected for thin composites [15]. On the impact site, only a small indentation (Figure 6a) was visible. It should be noticed that the indentations on the top surface were very shallow, with a depth of about 0.15 mm and 0.12 mm for the indentations of the first and second impact events, respectively.

**Figure 7:**
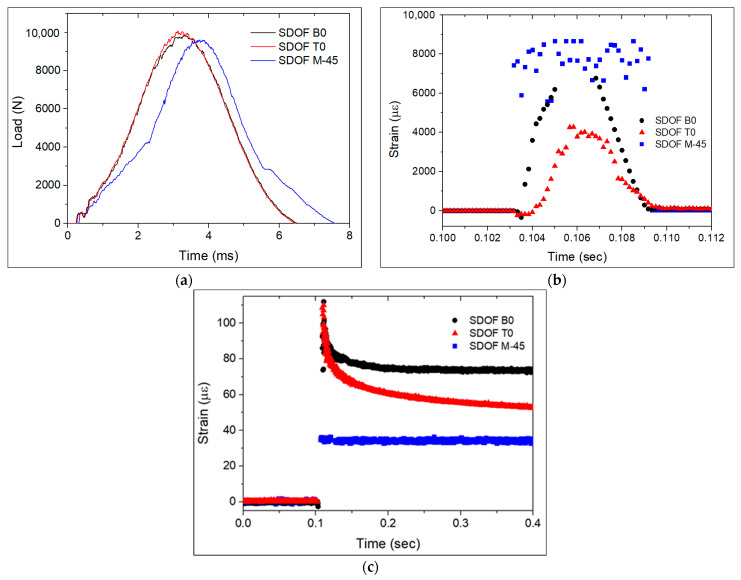
First impact with energy of 30.0 J on specimens with SDOF: (**a**) impactor load vs. time curves, (**b**) strain measured by the FBG_S+T_ at the moment of impact, and (**c**) developed residual strains.

Figure 7 presents the contact load measured by the impact setup and strain measured by the FBG sensors in the SDOF during the first impact with an energy of 30.0 J. The strain was null just before the impact and at around 0.1 s the impact happened on each sample. The FBG sensors measured a sudden increase and decrease in strain during impact, lasting about 6 ms, from 0.103–0.109 s. The impact duration was very close to the one measured by the impact setup (Figure 7a). This response can be attributed to the applied shear and compressive stresses at the time of impact. The through-thickness compressive stresses on the FBG sensor extended its length, resulting in a tensile strain. This fast-changing strain curve in Figure 7b can be very helpful in real-life situations to identify the nature of the event causing damage. Following the fast strain changing event, for the case of the specimens with OF at the B0 and T0 locations, the strain slowly reached a residual strain value (Figure 7c). For the case of the specimen with OF at the M-45 location, the strain quickly reached a residual strain value after the sudden increase in strain upon impact, which can be explained by the lower displacement of the layers in the mid-plan where this OF was embedded.

**Figure 8:**
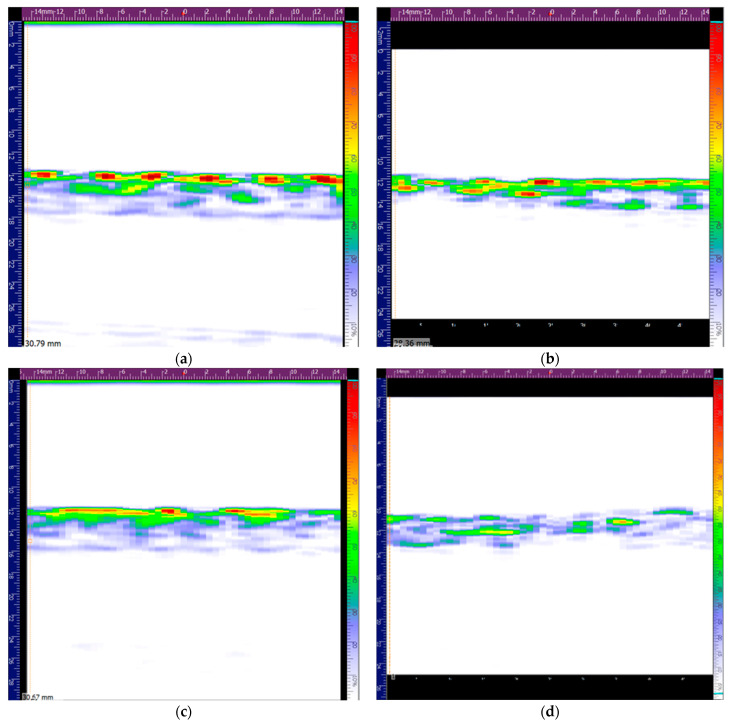
Phased array ultrasonic analysis of SDOF in location B0 prior to (**a**) and after (**b**) the first impact and of SDOF in location M-45 prior to (**c**) and after (**d**) first impact.

The shape of impactor contact load curve of the specimen with the SDOF at the M-45 location in Figure 7a reveals that this sample suffered the highest damage extent, which was confirmed by phased array ultrasonic observations (Figure 8). The analysed material corresponds to the middle colourful stripe, where the red colour in the top of the scale corresponds to the highest sound wave reflection, as the sound interacted with the laminate, whereas the lower part of the scale with the purplish colours corresponds to the lowest sound reflection and highest sound wave attenuation. A rectangular pattern was clear on the top surface of the laminates, which was due to the weft fibres in the CF fabric. The higher damage extent is visible in Figure 8d, with a whitish lower surface of the laminate. It should be noted that if one were to only evaluate the absolute values of strain measured by the FBG sensors, one could never guess that the sample with the OF in location M-45 was the most damaged one, since the SDOF in the M-45 location showed the lowest absolute residual strain value. The high extent of damage explains why this FBG sensor did not measure a strain value close to zero.

**Figure 9:**
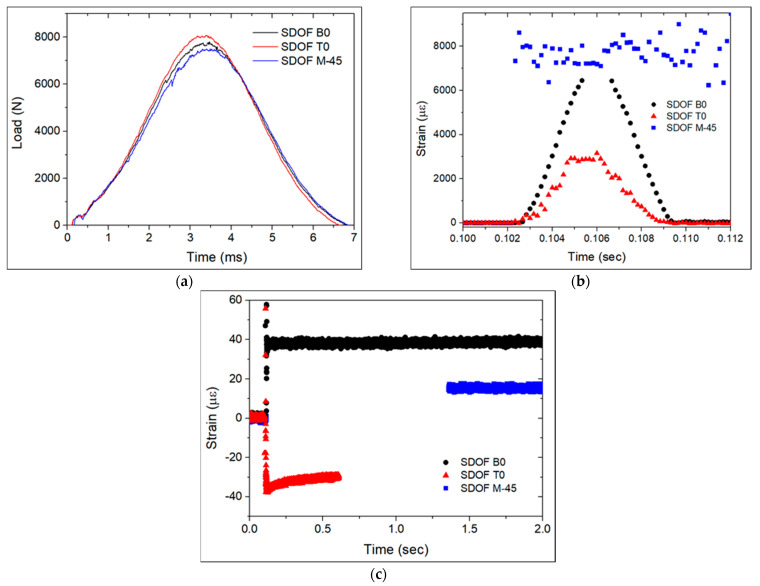
Second impact with an energy of 20.0 J on specimens with SDOF: (**a**) impactor load vs. time curves, (**b**) strain measured by the FBG_S+T_ at the moment of impact, and (**c**) developed residual strains.

Again, during the second impact, the FBG sensors measured a sudden increase and decrease in strain in the specimens with OF at the B0 and T0 locations (Figure 9b). Unexpectedly, the specimen with OF at the M-45 location kept its strain approximately constant at around 8000 με and it only decreased to about 15 με 1.2 s after the impact, as can be seen in Figure 9c. The sample with FBG sensors in the B0 location measured a tensile strain of about 38 με, whereas the sample with an FBG sensor in the T0 location measured a compressive strain of about −30 με. The FBG sensor in the M-45 location measured a rather lower residual tensile strain of about 15 με. The measured residual strains confirm the typical behaviour of a thin laminate under impact. Damage was mostly observed in the bottom plies due to the imposed bending stresses [15], resulting in a compressive strain in the FBG in the T0 location, a tensile strain in the FBG in the B0 location, and a rather lower residual strain in the FBG in the M-45 location. Chambers et al. [6] also reported the measurement of residual tensile strain by FBG sensors embedded below the mid-plan of CFRP after being subjected to LVI. The residual strains measured during the first impact with a higher energy revealed a different behaviour of the laminate. The strain measured by the SDOF at the B0 location was indeed a tensile strain, of about 75 με. However, the SDOF at the T0 and M-45 locations also measured tensile strains of about 48 and 34 με, respectively. The higher impact energy of 30.0 J caused a stiffer response in the laminate, with the dynamic load producing a rather complex strain-state condition with shear stresses prevailing over bending stresses. The second impact, at least for the case of the samples with OF in the B0 and M-45 locations, produced residual strain values of approximately half of the values observed in the first impacts with energies of 30.0 J.

**Figure 10:**
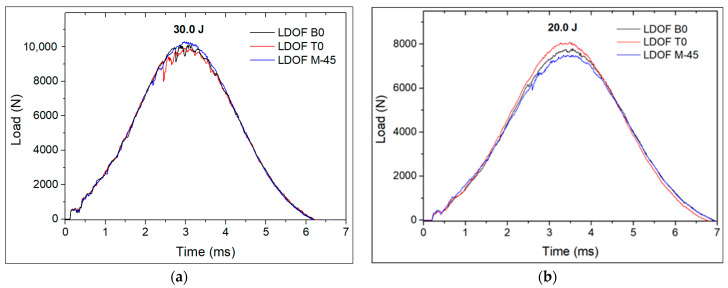
Impactor contact force for LDOF during the (**a**) first impact with 30.0 J and (**b**) second impact with 20.0 J.

The measured residual strains on specimens with LDOF at each OF through-thickness location after the first and second impacts are presented in Table 2. Impactor contact load curves (Figure 10) show that the higher damage extent during the first impact was imposed in samples B0 and T0, whereas sample M-45 had more damage extent during the second impact, where critical loads (*P_cr_*) were clearly observed by a sudden decrease in load. Due to technical issues with the interrogator equipment, it was not possible to acquire the real-time *λ_B_* variation of the FBG sensors. Instead, the full FBG spectra were recorded both prior to and after impact, from which the *λ_B_* was taken and used to calculate the imposed strain by the impact damage. The lower residual strain values measured on the first impacts, compared to the values measured by the SDOF, are explained by the larger distance between the impact sight and the FBG sensor. The LDOF in locations T0 and B0 measured approximately the same magnitude of residual strain, whereas the LDOF in location M-45 measured a lower residual strain. The second impacts, which were imposed closer to the FBG sensor, measured higher strain values. The observed behaviour was similar to that reported above for the laminates with embedded SDOF.

**Figure 11:**
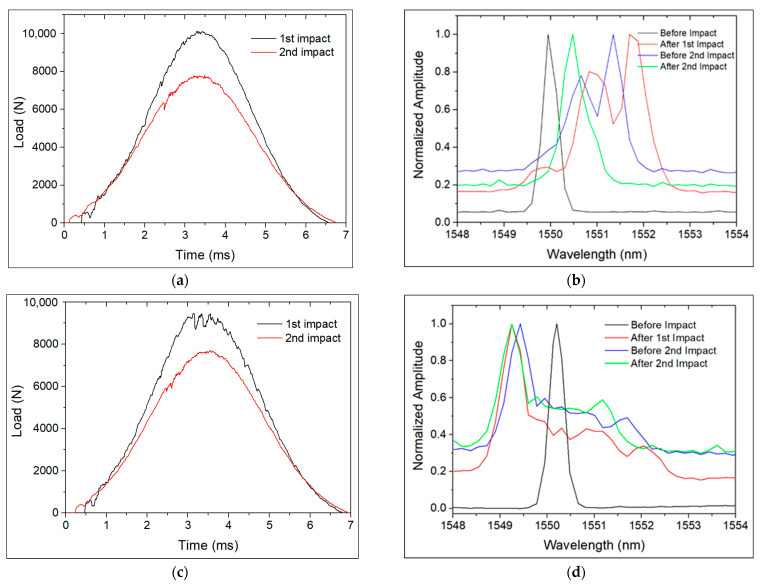
First (30.0 J) and second (20.0 J) low-velocity impacts on specimens with an LDOF with a Scheme 0 (**a**) and 002 (**c**), respectively, and (**b**), (**d**) corresponding changes on the FBG spectra.

Two samples with LDOF with a single FBG_S+T_ sensor embedded at each studied through-thickness OF location were also produced, where the FBG_S+T_ was in the central area of the impact sample, just below the impact site. These samples were also subjected to impacts with energies of 30.0 J and 20.0 J. The *λ_B_* of the FBG in location T0, just one CF layer away from the impactor surface, became broader and with more peaks (Figure 11b,d), whereas more severe damage was only observed in the first impact performed on sample 002 (Figure 11c). This reveals that a non-uniform strain was applied along the grating of the FBG, as reported elsewhere [16,17,18]. These sensors will likely fail to measure the actual strain condition in the FBG sensor and to detect impacts that may follow. The sample had the same mechanical condition on the “After 1st impact” and “Before 2nd impact” spectra. The slight wavelength shift measured between these spectra was due to small differences in sample temperature, as those spectra were taken on different days. Like in the previous experiments, the second impact with an energy of 20.0 J produced a compressive strain in the FBG sensors in the T0 location.

**Figure 12:**
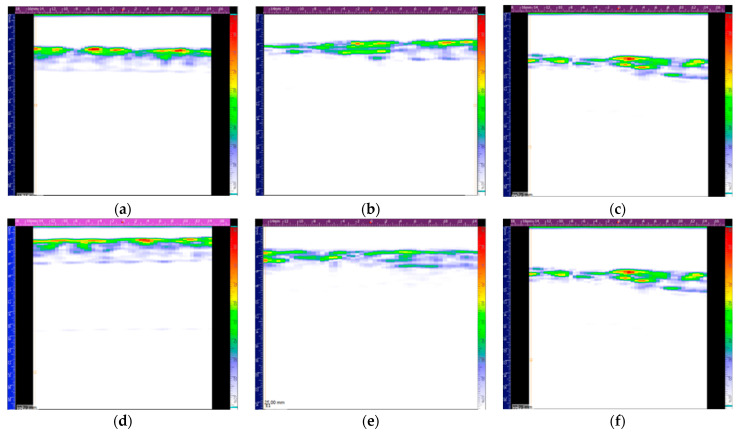
Phased array ultrasonic observations of specimens with an LDOF 001 (top) and 002 (bottom) with a single FBG_S+T_ in location T0: (**a**,**d**) before impact, (**b**,**e**) after the first impact, and (**c**,**f**) after the second impact.

After the first impact, the FBG sensor on LDOF 001 showed a peak split with two maximums at 1550.66 and 1551.35 nm. After the second impact, the FBG sensor showed again only one maximum at 1550.48 nm, which might indicate that the non-uniform strain was no longer applied. The first impact put the FBG under tension, whereas the second impact compressed the FBG, although it still showed a *λ_B_* above the initial condition without damage. Comparing to the original *λ_B_* of the FBG sensor, 1549.95 nm, a tensile strain of 434 με was introduced. After the first impact, the FBG sensor on LDOF 002 showed a very broad peak with a bandwidth of about 3.5 nm, with a main peak at 1549.43 nm. After the second impact, the FBG sensor showed again a broad peak with about the same bandwidth. Comparing the main peaks in the spectra prior to and after the second impact, a compressive strain of −147 με was introduced during the second impact. However, the spectra after each impact showed the same *λ_B_*. A strain of −720 με was measured after the first impact. Looking at Figure 11c, the first impact did in fact produce more damage than the second one. The phased array ultrasonic observations in Figure 12 show very similar damage between the two samples and it is possible to see that further damage was introduced in the second impacts, as both impacts were made about 1 cm away.

Samples with LDOF in locations M-45 and B0 did not have their FGB peak shape change, even though the FBG sensors were also located below the impactor site, but at lower thickness levels in the laminate. These sensors read much higher strain values (Table 3) compared to the other samples with FBG sensors further away from the impact site. Again, the sample with LDOF in location B0 measured a higher residual strain value than the sample with LDOF in the neutral axis. The FBG sensor of the sample with LDOF in location B0 broke during the second impact of 20.0 J. As mentioned previously, thin laminates had most of the damage imposed in the bottom plies.

One sample of each condition, with a 2-FBG sensor array on SDOF and LDOF at each studied location, was further exposed to eight more consecutive impacts, all in the same location. Samples with SDOF were exposed to impacts with energies of 20.0 J, as FBG_S+T_ sensors were about 2–3 cm away from the impact location, and the samples with LDOF were exposed to impacts with energies of 25 J, as the FBG_S+T_ sensors were about 4–5 cm away from the impact location. All FBG_S+T_ sensors survived the 10 total impacts, but two temperature-sensitive FBG sensors were damaged between the third and fifth impacts. For the samples with OF in locations B0 and T0, the third impact followed the same behaviour observed in the second impact: the FBG_S+T_ in B0 location measured residual tensile strains, whereas the FBG_S+T_ in location T0 measured residual compressive strains. In most of the samples, the fourth to 10th impacts produced either tensile or compressive strains due to accumulated damage by the precedent impacts in that same location.

### 3.3. Comparison of Low-Velocity-Impact Resistance between CFRP Laminates Embedded with Small- and Large-Diameter Optical Fibres

The percentage of absorbed energy and *P_cr_* were compared among the instrumented laminate specimens, with the aim to evaluate whether instrumentation with SDOF or LDOF would impair the impact resistance of the laminates. As can be seen in Table 4, the non-instrumented reference laminates showed lower absorbed impact energy than the instrumented laminates, which reveals that the embedded OF contributed to a higher level of damage in the composite specimens. However, the difference between non-instrumented and instrumented laminates was only about 5%. Moreover, it should be noted that each specimen of reference laminate was impacted only once; hence, the specimens impacted with an energy of 20.0 J were not previously impacted with an energy of 30.0 J, unlike the instrumented laminates. All LDOF-containing specimens showed a *P_cr_* in the first impact (Figure 10a), whereas only SDOF in the M-45 location showed a *P_cr_* (Figure 10b). Strong evidence of the advantage on the use of SDOF over LDOF was not observable. The almost negligible decline of impact properties, in comparison to the non-instrumented laminate, can be overcome by composite design optimisation to take full advantage of FBG sensors for BVID detection.

## 4. Conclusions

Different through-thickness embedding locations of FBG sensors in CFRP laminates were studied for BVID detection. The higher energy of the first impact (30.0 J) caused a stiffer response in the CFRP, with FBG sensors in the T0 and B0 locations measuring residual tensile strains of similar amplitude, about 50–75 με for samples with SDOF and 6–8 με for samples with LDOF. Rather lower residual strain values were measured by the FBG sensors in the mid-plan, about 35 με in the SDOF and −1 με in the LDOF. Bending of the laminate was revealed during the second impact with lower energy (20.0 J) by the residual tensile strain in the bottom layers (38 με in the SDOF and 35 με in the LDOF) and residual compressive strain in the top layers (−30 με for samples with SDOF and −26 με for those with LDOF). Besides the residual strain produced by the impact event, the FBG sensors identified the dynamic response of the CFRP with strain peaks, reaching up to 8000 με at the time of impact. This is particularly useful for the identification of the damage source in real-life applications, enabling improved damage severity assessment. For BVID detection in thin laminates, it is recommended that OF be embedded close to the bottom layers, where the damage condition can be more reliably evaluated, with FBG sensors being closer to the damaged layers. Impacts performed on samples with embedded FBG in the T0 location, right under the impactor area, produced non-uniform strain, which hindered the calculation of the applied strain, and FBG in the M-45 location measured smaller strain values, which may have misled the evaluation of the damage condition.

For the studied density of embedded OF, the impairment of impact resistance was minimal when compared to the reference laminates. Furthermore, there was no evident advantage of the use of SDOF over LDOF, since the impacts produced comparable levels of absorbed energy in the instrumented laminates.

## Figures and Tables

**Table 1:** Absorbed energy and maximum impactor contact force for each level of impact energy performed on the reference CFRP.

Impact Energy (J)	Absorbed Energy (%)	Max. Impact Force (N)
13.1	45.5 ± 0.3	5662 ± 15
15.1	45.4 ± 0.4	6317 ± 35
17.5	46.2 ± 0.1	6986 ± 24
20.0	41.1 ± 0.3	7696 ± 50
25.0	46.5 ± 0.7	9069 ± 24
30.0	44.8 ± 0.6	9899 ± 85
40.1	54.0 ± 3.2	12189 ± 39

**Table 2:** Residual strains measured by embedded LDOF in each of the studied through-thickness locations after the first and second impacts.

Sample	Strain (με)	
	1st Impact (30.0 J)	2nd Impact (20.0 J)
LDOF T0	8	−26
LDOF M-45	−1	−16
LDOF B0	6	35

**Table 3:** Residual strains measured by embedded LDOF in each of the studied through-thickness locations after the first and second impacts. The impact location was on top of the FBG sensors.

Sample	Strain (με)	
	1st Impact (30.0 J)	2nd Impact (20.0 J)
LDOF M-45	173	−8
LDOF B0	519	(FBG destroyed)

**Table 4:** Percentage of absorbed energy and critical load values measured on impacts with 20.0 and 30.0 J.

Sample	Absorbed Energy (%)		*P_cr_* (N)	
	30.0 J	20.0 J	30.0 J	20.0 J
LDOF T0	50.5	46.7	9148	−
LDOF B0	49.0	47.9	9952	−
LDOF M-45	47.9	48.0	8032	−
Average	49.1 ± 1.1	47.5 ± 0.2	9044 ± 787	−
SDOF T0	49.3	42.3	−	−
SDOF B0	49.9	45.9	−	6160
SDOF M-45	50.8	48.7	4255	6400
Average	50.0 ± 0.6	45.6 ± 2.6	−	6280 ± 120
Reference laminate	44.8 ± 0.6	41.1 ± 0.3	8015 ± 58	−

## Data Availability

Data are contained within the article.
